# Cohort profile update: the Korean Cancer Prevention Study-II (KCPS-II) biobank

**DOI:** 10.4178/epih.e2025040

**Published:** 2025-07-29

**Authors:** Heejin Kimm, Keum Ji Jung, Wes Spiller, Yeun Soo Yang, So Young Kim, Min Young Park, Sun Mi Lee, Sun Ha Jee

**Affiliations:** 1 Department of Epidemiology and Health Promotion, Graduate School of Public Health, Yonsei University, Seoul, Korea; 2 Institute for Health Promotion, Graduate School of Public Health, Yonsei University, Seoul, Korea; 3 Health Insurance Research Institute, National Health Insurance Service, Wonju, Korea; 4 JS Link, Inc., Seoul, Korea

**Keywords:** Biological specimen banks, Cohort studies, Epidemiology, Genetic epidemiology

## Abstract

Chronic diseases such as cancer and cardiovascular disease have a substantial impact on mortality and global disease burden. The Korean Cancer Prevention Study-II (KCPS-II) biobank was established to investigate these chronic diseases, with a particular focus on metabolic risk factors. Recently, genetic information reflecting diverse ancestries has been incorporated to support a precision medicine approach. These data can be leveraged to identify variation in causal effects among different ancestral groups, thereby informing the development of more effective, ancestry-specific treatments. From 2004 to 2013, baseline data were collected from 156,701 individuals aged 20-85 years, recruited from 18 health promotion centers across Korea. Outcome data are routinely obtained from Statistics Korea (mortality data), the National Cancer Center (cancer registry data), and the National Health Insurance Service (morbidity data). Additionally, new participants have been enrolled since 2022 as part of an ongoing expansion. This population-based cohort, enriched with genetic data, provides a robust foundation for research aimed at elucidating causal relationships in chronic disease.

## Key Message

• The Korean Cancer Prevention Study II (KCPS-II) Biobank comprises 156,701 adults aged 20–85 years, recruited from 18 health examination centers nationwide.

• Baseline data include socioeconomic, medical history, behavioral, physiological, and blood sample information, with completed genetic analyses.

• KCPS-II is an ongoing cohort with follow-up through linkage to secondary data on mortality, cardiovascular events, and cancer incidence.

• This resource enables the integration of epidemiological and genetic data to investigate disease mechanisms and provide scientific evidence for prevention and control in the Korean population.

## INTRODUCTION

The Korean Cancer Prevention Study-II (KCPS-II) biobank was initially established as a resource for investigating outcomes related to cancer and cardiovascular disease, with a particular emphasis on metabolic risk factors. At baseline, phenotypic data were collected via self-report questionnaires and health examinations, including anthropometric, demographic, and medical history information. Approximately 46% of the sample returned for re-examination, while follow-up data on mortality and hospitalization were obtained through linkage with national health records. This paper provides an update to the previous profile of the KCPS-II cohort, which comprises 156,701 participants aged 20-85 years recruited from 18 health promotion centers across Korea, and includes information on additional data collection and follow-up [1].

The need for large-scale cohorts with genetic information from diverse ancestries has been widely recognised within the epidemiological community [2]. Such data enable the investigation of causal effect variation among ancestry groups, supporting precision medicine strategies that prioritise ancestry-specific, evidence-based interventions. Furthermore, genetic analyses often require large sample sizes to achieve adequate statistical power, frequently necessitating meta-analyses to aggregate evidence [3]. In response to these needs, comprehensive genotyping has been performed for all KCPS-II participants.

**Figure 1.**
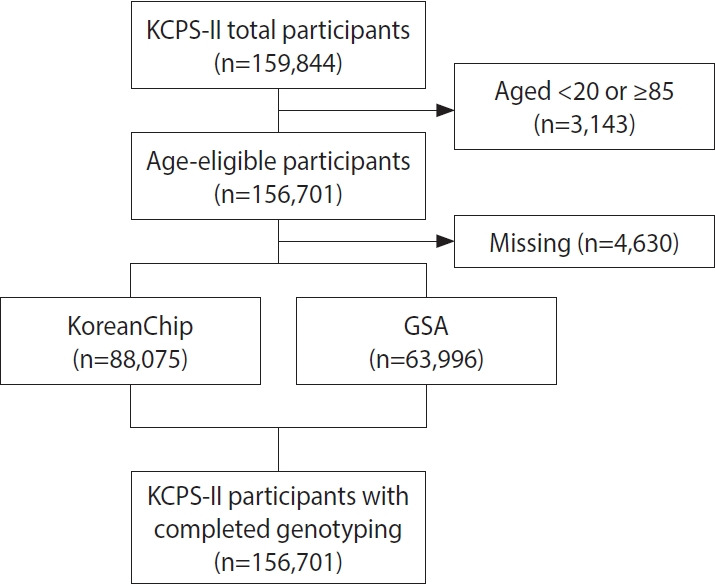
Korean Cancer Prevention Study-II (KCPS-II) genotyping flow diagram. KoreanChip, Korea Biobank Array; GSA, Global Screening Array.

This study update differs from the previous cohort profile in 3 key respects. First, from 2015 to 2022, genotyping was completed for the entire KCPS-II cohort. In 2016, genotyping of 5,000 individuals using the Korea Biobank Array (KoreanChip) version 1.0 was performed as part of a case-cohort study [4]. Subsequently, an additional 8,855 individuals were genotyped, resulting in a dataset of 13,855 individuals [5,6]. Genotyping was later expanded to include 88,075 individuals using KoreanChip data and 63,996 individuals using the Global Screening Array (GSA) chip, yielding genotype data for 152,071 individuals to date (Figure 1). Second, we report updated follow-up data for morbidity and mortality, particularly with respect to cancer and cardiovascular disease.

With the availability of genotype data, analyses have expanded to include genome-wide association studies and Mendelian randomization (MR). Additional recent assessments include faecal sampling for microbiome studies and metabolomics for more detailed molecular phenotyping. Air pollution exposure data are also being updated to facilitate research on the health effects of air pollution. Mortality data are obtained annually from Statistics Korea, cancer registry data from the National Cancer Center (NCC), and morbidity data from the National Health Insurance Service (NHIS) to provide continuous updates on participants’ health status. Third, since 2022, additional participants have been recruited, with biosamples (including informed consent and blood samples) collected. As of January 31, 2025, a total of 7,579 new participants have been enrolled.

## STUDY PARTICIPANTS

The cohort includes data from 156,701 participants (94,840 men and 61,861 women) recruited from 18 health promotion centers throughout Korea (Supplementary Material 1). Recruitment occurred from 2004 to 2013, enrolling participants aged 20-84 years. Of these centers, only Yonsei University Shinchon Severance Hospital has enrolled an additional 7,579 participants as of January 31, 2025. Participants who did not provide informed consent or blood samples were excluded from the KCPS-II cohort. The general characteristics of the total study population (n=164,280), which includes the 7,579 newly enrolled individuals in addition to the original 156,701, are summarized in Table 1.

### Ethics statement

The study protocol for the original cohort profile was approved by the Institutional Review Board (IRB) of Severance Hospital (approval No. 4-2011-0277). Recruitment of the 7,579 newly added participants was approved under a separate IRB protocol (approval No. 4-2022-1262). All participants provided written informed consent prior to participation, in accordance with the Declaration of Helsinki.

## MEASUREMENTS

### Morbidity and mortality follow-up

Since the completion of study recruitment in 2013, follow-up data on morbidity and mortality have been updated annually. Morbidity data are sourced from 2 institutions: the NCC for cancer diagnoses and the NHIS for non-cancer diagnoses. Data from the NCC include International Classification of Disease, 10th revision (ICD-10) codes for cancer type, date of diagnosis, histological type, and Surveillance, Epidemiology, and End Results stage. Table 2 presents statistics related to all-cause mortality, cancer-related illness, and non-cancer-related illness as defined by ICD-10 codes. As shown in Table 2, the number of confirmed cancer cases increased approximately 2.4-fold between 2014 and 2021, rising from 5,669 to 14,469 cases, with especially notable increases in cervical and prostate cancers.

Data on non-cancer-related illnesses, derived from the NHIS, include records of hospitalizations, outpatient visits, and prescription histories for all KCPS-II participants. In line with the original KCPS-II cohort profile, we provide an updated table with information on ischemic heart disease (IHD), stroke, diabetes, end-stage renal disease, dementia, chronic obstructive pulmonary disease, and tuberculosis. During the period from 2015 to 2022, cases of IHD, stroke, and diabetes have more than doubled. Mortality data, including date and cause of death, show an increase from 1,184 to 3,872 deaths between 2015 and 2021. Of the deceased, 2,856 were men and 1,016 were women. Among these, 59.4% (1,696 individuals) died from cancer, while 599 deaths were attributed to cardiovascular or cerebrovascular diseases.

### Genotyping of the Korean Cancer Prevention Study-II cohort

The genotyping of the KCPS-II cohort was conducted following a standardized protocol for quality control and genotype imputation. Before expanding genotyping to the entire KCPS-II sample, an initial subset of 14,591 participants was genotyped using the KoreanChip 1.0 array [7]. Subsequent genotyping was carried out using a combination of the GSA 2.0 and KoreanChip 1.1 arrays. After excluding participants with missing genotyping data, the final sample comprised 156,701 individuals genotyped with 3 arrays: 63,996 (40.8%) using the GSA 2.0 chip and 88,075 (56.2%) using KoreanChip 1.0 and 1.1 arrays (Supplementary Material 2).

The use of multiple arrays was motivated by improvements in variant detection capacity with the KoreanChip 1.1, which became available after the genotyping process had already begun. Table 3 presents the number of markers directly measured by each array, with KoreanChip 1.1 featuring approximately 12% more markers than the GSA chip. Importantly, data obtained with KoreanChip 1.0 were integrated with KoreanChip 1.1 data to create a combined KoreanChip dataset containing markers common to both arrays.

For participants genotyped with GSA or KoreanChip 1.1, quality control procedures included the exclusion of individuals with gender mismatches, violations of Hardy-Weinberg equilibrium (p<5×10^-4^), or a missingness threshold exceeding 5% [8]. Additionally, variants were excluded before imputation if their allele frequency differed by more than 20% compared to the 1000 Genomes Phase 3 East Asian sample [9]. Variants with a minor allele frequency below 5% or a call rate below 95% were also removed (Supplementary Material 3). To assess population stratification and batch effects, principal component analysis (PCA) was conducted in 2 stages using the flashpca software tool [10]. Initially, PCA was used to examine stratification within the non-overlapping GSA and KoreanChip subsets, using up to 10 principal components. Each cluster exhibited a normal distribution with no distinct group characteristics, indicating minimal population stratification—likely reflecting the genetic homogeneity of the Korean population (Supplementary Material 4).

Potential batch effects arising from the use of multiple genotyping arrays were further evaluated by performing PCA on all participants genotyped with either the GSA or KoreanChip arrays (Supplementary Material 5). The number of markers common to all arrays was initially 94,735, which was then reduced to independent variants (r2<0.2) that satisfied previously described criteria. This resulted in a set of 26,165 markers for PCA, with no evidence of discernible chip-to-chip batch effects.

**Figure 2.**
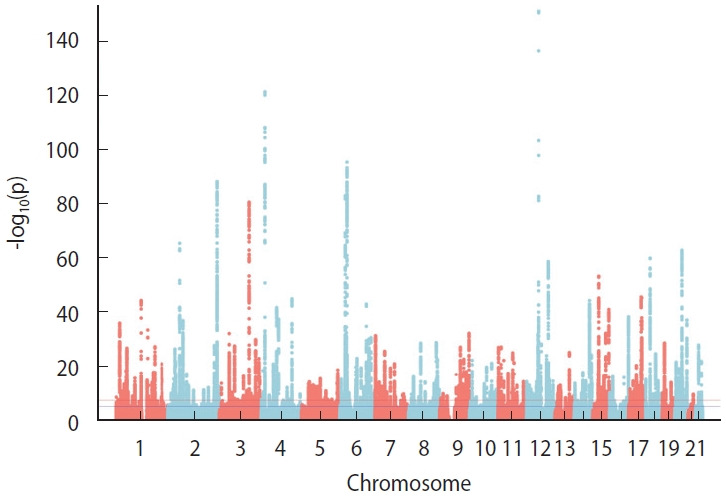
Manhattan plot of height.

Using the combined set of markers from the GSA and KoreanChip arrays, genotype imputation was performed with IMPUTE v5 and the East Asian subset of the 1,000 Genomes Reference Panel. Applying an info score threshold of ≥0.8 yielded a final genetic dataset containing 7,392,216 variants for all genotyped individuals [11]. For the final set of study participants who passed genetic quality control, an example Manhattan plot was generated using PLINK analysis of height (Figure 2).

## KEY FINDINGS

Previous research utilising KCPS-II data can be broadly categorised into genetic and metabolomic studies. Here, we provide a summary of key findings in each field. Findings published after 2022 are summarised in Supplementary Material 6.

### Studies leveraging Korean Cancer Prevention Study-II genetic data

The KCPS-II cohort has served as a valuable resource for investigating the causal mechanisms underlying cardiovascular disease, employing a range of analytical approaches. For example, Jung et al. [12] evaluated the predictive accuracy for stroke risk using a traditional risk score, a genetic risk score, and a combination of both genetic and traditional risk factors. They observed that the relative performance of each scoring method varied by participant age, with traditional risk factors having greater influence than genetic factors in individuals over 40. Notably, the most effective approach combined both genetic and traditional risk factors, outperforming either model alone, regardless of age.

Cardiovascular disease has also been explored in the KCPS-II cohort using MR. Using individual-level data, Lee et al. [4] found that the observed association between serum bilirubin and stroke in observational analyses could be due to unmeasured confounding, as the association disappeared when applying MR methods. However, 2-sample MR approaches using both KCPS-II and Korean Genome and Epidemiology Study (KoGES) data suggest a potential inverse association between serum bilirubin and stroke risk. Choi et al. [13] reported that this association was strongest for ischemic stroke, suggesting that previous analyses may have been underpowered.

Further MR studies with KCPS-II data have examined the roles of serum lipid levels and bilirubin as potential risk factors for IHD. In a 2-sample MR analysis with KoGES data, Jeon et al. [5] found no association between serum bilirubin and IHD.

KCPS-II-based cancer research using genetic data has primarily focused on the causal mechanisms of colorectal cancer. Jung et al. [14] investigated the impact of integrating genetic data into colorectal cancer risk prediction models, finding the most substantial improvements in predicting rectal cancer alone. Their results suggest that modelling colon and rectal cancers separately may be more effective in the Korean population. Park et al. [15] identified T-cadherin as a potential risk factor for colorectal cancer using a gene-gene interaction analysis, supporting an interaction between the CHD13 and APN genes. Research by Lee et al. [16] further suggested an interaction between genetic variant rs7930 and miRNA miR-4273-5p as a causal pathway for colorectal cancer [16]. Additionally, Jung et al. [17] found that while both fasting glucose and genetic factors contribute to colorectal cancer development, they did not appear to act synergistically.

Several studies of colorectal cancer have involved collaboration with other large-scale genetic cohorts. For example, Lu et al. [18,19] identified 3 novel loci for colorectal cancer by conducting a genome-wide association study (GWAS) with 23,572 colorectal cancer patients and 48,700 controls of East Asian ancestry, and also found evidence for additional risk variants in previously identified loci. GWAS using KCPS-II data, specifically among East Asian populations, have also discovered novel loci and variants for waist-to-hip ratio, waist circumference, and fasting glucose [20,21]. KCPS-II data have contributed to trans-ancestry analyses as well, such as Spracklen et al. [22], who identified new loci influencing cholesterol and triglyceride levels in both East Asian and European populations.

MR analyses have examined the effects of alcohol consumption on potential disease risk factors. Jee et al. [23,24] investigated the impact of alcohol consumption on serum glucose levels and hyperuricemia, finding positive associations with both outcomes. Their work also adds to the body of evidence for a gene-by-environment interaction involving the *ALDH2* gene and participant gender. Historically, differences in alcohol consumption by gender in Korea meant that the influence of *ALDH2* was primarily observed in men. Such interactions have inspired a subfield of MR research focusing on gene-by-environment effects; however, it is important to note that gender differences in alcohol consumption in Korea have decreased substantially in recent years [25]. A recent study also explored the causal relationship between genetically determined alcohol consumption and cancer risk, providing evidence that the amount of alcohol consumed is causally linked to the risk of rectal cancer and liver cancer in hepatitis B surface antigen-negative individuals [26]. GWAS of 36 traits in the KCPS-II biobank and meta-analyses across Asian and UK biobanks identified 301 novel loci and 4,588 additional loci, revealing population-specific architectures and an East Asian–specific pleiotropic ALDH2 variant [27].

### Studies leveraging Korean Cancer Prevention Study-II metabolite data

The availability of detailed metabolomic data in the KCPS-II cohort has enabled a wide range of studies, primarily investigating liver function and cancer-related outcomes. In a longitudinal analysis over a 7-year follow-up period, Yoo et al. [28] identified 9 metabolic pathways associated with the onset of liver cirrhosis, including myristic acid, palmitic acid, linoleic acid, eicosapentaenoic acid, lysophosphatidic acid (18:1), glycolic acid, lysophosphatidylcholine (22:6), and succinylacetone. Choi et al. [6] also found inverse associations between bisphenol and phthalate metabolites and serum bilirubin, suggesting a potential link to liver function within the Korean population.

The role of metabolites in carcinogenesis has also been investigated for breast, prostate, and pharyngolaryngeal cancers. In a prospective study, Yoo et al. [29] found ten metabolic pathways that differed significantly between 84 breast cancer cases and 88 controls, including pathways related to amino acid metabolism, arachidonic acid metabolism, fatty acid metabolism, linoleic acid metabolism, and retinol metabolism. In the context of men pharyngolaryngeal cancer, Jee et al. [30] identified oleamide, tryptophan, and linoleyl carnitine as potential biomarkers in a case-control study, while Khan et al. [31] highlighted tryptophan, kynurenine, and anthranilate as potential biomarkers for prostate cancer.

## STRENGTHS AND WEAKNESSES

The increase in genetic data available from KCPS-II is likely to yield substantial benefits for research focusing on populations of East Asian ancestry. With the genotyping of entire participant cohorts, genetic analyses now have greatly increased power to detect causal effects and support complex analyses that were previously infeasible. Moreover, because most Korean citizens are enrolled in national health insurance, it is possible to obtain detailed information on a wide array of health outcomes through record linkage. Consequently, the KCPS-II cohort makes a significant contribution to the growing body of resources helping to address disparities in genetic research representation.

As with many population-based cohorts, analyses using KCPS-II data may be subject to selection bias at recruitment. Participants were mostly recruited during health checkups and were not generally patients; in many cases, participation was largely subsidised by companies for their employees and family members. As a result, the sample may not be fully representative of the entire Korean population. Bias related to the early detection of diseases, prior to the manifestation of symptoms, must also be considered, especially with regard to changes in health behaviours that may result from early identification of chronic disease. Furthermore, until genotyping of the entire sample using KoreanChip 1.1 is complete, analyses are limited to imputed variants shared by both the GSA and KoreanChip arrays.

## DATA ACCESSIBILITY

Access to data from the KCPS-II cohort is not publicly available, but summary statistics and further information may be requested. The study group has collaborated with several other groups to share data and promote new collaborations. Potential collaborators are invited to contact the corresponding author (Sun Ha Jee, e-mail: jsunha@yuhs.ac) at the administrative office of the KCPS-II biobank, Institute for Health Promotion, Graduate School of Public Health, Yonsei University, Seoul, Korea.

## Figures and Tables

**Table 1.** General characteristics of the study population (n=164,280), including 7,579 newly added participants

Characteristics	n (%)	Mean±SD
Demographic variable		
Age	164,280 (100)	42.0±10.8
Gender (women, %)	164,280 (100)	39.8
Physiological characteristics		
Anthropometry		
Weight	163,839 (99.7)	65.9±12.1
Height	163,835 (99.7)	166.8±8.4
Waist circumference	158,742 (97.0)	80.7 (9.7)
Blood pressure		
Systolic blood pressure	161,268 (98.0)	118.0±14.3
Diastolic blood pressure	161,268 (98.0)	74.3±10.2
Blood measurements		
Lipid profile		
Total cholesterol	163,641 (99.6)	189.4±34.2
HDL cholesterol	161,864 (98.5)	52.6±11.5
LDL cholesterol	162,575 (99.0)	112.6±53.3
Triglyceride	162,992 (99.2)	134.5±91.5
Liver function test		
GOT	163,663 (99.6)	23.3±17.2
GPT	163,665 (99.6)	25.2±25.5
GGT	162,855 (99.1)	35.9±50.5
Kidney function		
Creatinine	155,844 (94.9)	0.98±0.24
BUN	156,454 (95.2)	13.8±3.7
Tumour markers		
CA125	47,891 (29.2)	10.8±17.9
CEA	140,576 (85.6)	1.8±1.8
CA19-9	102,245 (62.2)	9.6±17.9
Others		
Albumin	153,305 (93.3)	4.5±0.3
Bilirubin	152,392 (92.8)	0.87±0.36
Uric acid	150,970 (91.9)	5.42±3.10

SD, standard deviation; HDL, high-density lipoprotein; LDL, low-density lipoprotein; GOT, glutamate oxaloacetate transaminase; GPT, glutamic pyruvic transaminase; GGT, gamma-glutamyl transferase; CEA, carcinoembryonic; BUN, blood urea nitrogen; CA125, carbohydrate antigen 125; CA19-9, carbohydrate antigen 19-9.

**Table 2.** Number of deaths, vascular events, and cancer incidents according to major ICD-10 categories over 15 years of follow-up in the Korean Cancer Prevention Study-II

	Men		Women		Total	
No. of participants	94,840	94,840	61,861	61,861	156,701	156,701
	**By 2015**	**By 2021**	**By 2015**	**By 2021**	**By 2015**	**By 2021**
Mortality						
Deaths	869	2,856	315	1,016	1,184	3,872
	**By 2015**	**By 2022**	**By 2015**	**By 2022**	**By 2015**	**By 2022**
Morbidity						
All vascular	4,908	8,747	2,344	3,804	7,252	12,551
Ischemic heart disease	2,191	5,451	741	1,858	2,932	7,309
Stroke	1,357	3,027	781	1,712	2,138	4,739
Ischaemic stroke	637	1,557	294	630	931	2,187
SAH	111	209	66	165	177	374
ICH	156	329	69	133	225	462
Other vascular	456	1,590	393	698	849	2,288
Diabetes^1^	4,521	14,178	1,828	5,648	6,349	19,826
End stage renal disease^1^	299	1,311	122	418	421	1,729
Dementia^1^	154	1,422	95	1,605	249	3,027
COPD^1^	318	1,866	82	672	400	2,538
Tuberculosis^1^	265	1,663	133	995	398	2,658
	**By 2014**	**By 2021**	**By 2014**	**By 2021**	**By 2014**	**By 2021**
All cancer	3,167	7,760	2,502	6,679	5,669	14,439
Thyroid	856	1,428	1,291	2,221	2,147	3,649
Stomach	523	1,332	139	3,423	662	1,755
Colorectal	371	871	148	356	519	1,227
Breast	3	8	429	1,396	432	1,404
Lung	230	709	88	264	318	973
Prostate	295	911	-	-	295	911
Liver	210	480	28	85	238	565
Kidney	103	334	21	90	127	424
Cervix	-	-	40	177	40	177

Since the secondary data linkage has not yet been completed for the entire population of 164,280, updated figures are presented for the 156,701 participants whose secondary data were linked up to 2022. SAH, subarachnoid haemorrhage; ICH, intracerebral haemorrhage; COPD, chronic obstructive pulmonary disease.

1 The number of outcomes up to 2015 was calculated using hospitalization data only, whereas the updated number of outcomes through 2022 was calculated using both hospitalization and outpatient data.

**Table 3.** Number of genetic variants according to the array chip

Variables	No. of variants		
	Original	After imputation	Info score≥0.8
Array chip information (merge)		81,169,748	7,392,216
Global Screening Array 2.0	728,094	81,278,852	8,115,034
KoreanChip (1.0)	833,536	84,087,333	8,834,111
KoreanChip (1.1)	827,783	84,151,324	8,726,326

KoreanChip, Korea Biobank Array.

## Supplementary materials

Supplementary Material 1. List of 18 health promotion centers included in KCPS-II

Supplementary Material 2. Genetic array chip information in the Korean Cancer Prevention Study-II

Supplementary Material 3. Quality Control process of KCPS-II Biobank

Supplementary Material 4. Principal Component Analysis of Non-overlapping Global Screening Array and KoreanChip Subsets

Supplementary Material 5. Principal Component Analysis to Assess Batch Effects Between Global Screening Array and KoreanChip Genotyping Arrays

Supplementary Material 6. The list of studies produced from KCPS-II Biobank using genomic or metabolomic data (after 2022)
