# Supplementary material for: Cohort profile update: the Korean Cancer Prevention Study-II (KCPS-II) biobank
Source: Epidemiol Health. 2025 Jul 29;47:e2025040. doi: 10.4178/epih.e2025040 (PMC12673288; doi:10.4178/epih.e2025040)
Supplement: Supplementary Material 1. — List of 18 health promotion centers included in KCPS-II [file epih-47-e2025040-Supplementary-1.docx]

**Supplementary Material 1. List of 18 health promotion centers included in KCPS-II**

| No | Name of center | Area |
| --- | --- | --- |
| 1 | Korea Univ. Guro Hosp. | Seoul |
| 2 | Ewha Womans Univ. Mokdong Hosp. | Seoul |
| 3 | Yonsei Univ. Shinchon Severenace Hosp. | Seoul |
| 4 | KMI Gwanghwamun | Seoul |
| 5 | Kyung Hee Univ. Hosp. | Seoul |
| 6 | Seoul Med. Center | Seoul |
| 7 | KMI Yeouido | Seoul |
| 8 | Yonsei Univ. Gangnam Severance Hosp. | Seoul |
| 9 | KMI Gangnam | Seoul |
| 10 | Asan Med. Center | Seoul |
| 11 | Catholic Univ. Bucheon ST. Mary’s Hosp. | Gyeonggi province |
| 12 | KMI Suwon | Gyeonggi province |
| 13 | SNU Bungang Hosp. | Gyeonggi province |
| 14 | Bundang Cha Hosp. | Gyeonggi province |
| 15 | Hanyang Univ. Guri Hosp. | Gyeonggi province |
| 16 | KMI Gwangju | Other areas |
| 17 | KMI Daegu | Other areas |
| 18 | KMI Busan | Other areas |

Hosp. =hospital, KMI=Korea Medical Institute, Med=Medical, SNU=Seoul National University, Univ.=University
