# Supplementary material for: Cohort profile update: the Korean Cancer Prevention Study-II (KCPS-II) biobank
Source: Epidemiol Health. 2025 Jul 29;47:e2025040. doi: 10.4178/epih.e2025040 (PMC12673288; doi:10.4178/epih.e2025040)
Supplement: Supplementary Material 2. — Genetic array chip information in the Korean Cancer Prevention Study-II [file epih-47-e2025040-Supplementary-2.docx]

**Supplementary Material 2. Genetic array chip information in the Korean Cancer Prevention Study-II**

|  | Men  (n=94 840) | Women  (n=61 861) | Total  (n=156 701) |
| --- | --- | --- | --- |
| Chip information |  |  |  |
| Global Screening Array 2.0 | 27 369 (28.9) | 36 627 (59.2) | 63 996 (40.8) |
| Korean Chip (1.0) | 9 580 (10.1) | 5011 ( 8.1) | 14 591 ( 9.3) |
| Korean Chip (1.1) | 55 200 (58.2) | 18 284 (29.6) | 73 484 (46.9) |
| No data | 2 690 ( 2.8) | 1 940 ( 3.1) | 4 630 ( 3.0) |

Values are n (%)
