# Supplementary figures and images for: Cohort profile update: the Korean Cancer Prevention Study-II (KCPS-II) biobank
Source: Epidemiol Health. 2025 Jul 29;47:e2025040. doi: 10.4178/epih.e2025040 (PMC12673288; doi:10.4178/epih.e2025040)

**Supplementary Material 3. Quality Control process of KCPS-II Biobank**

**
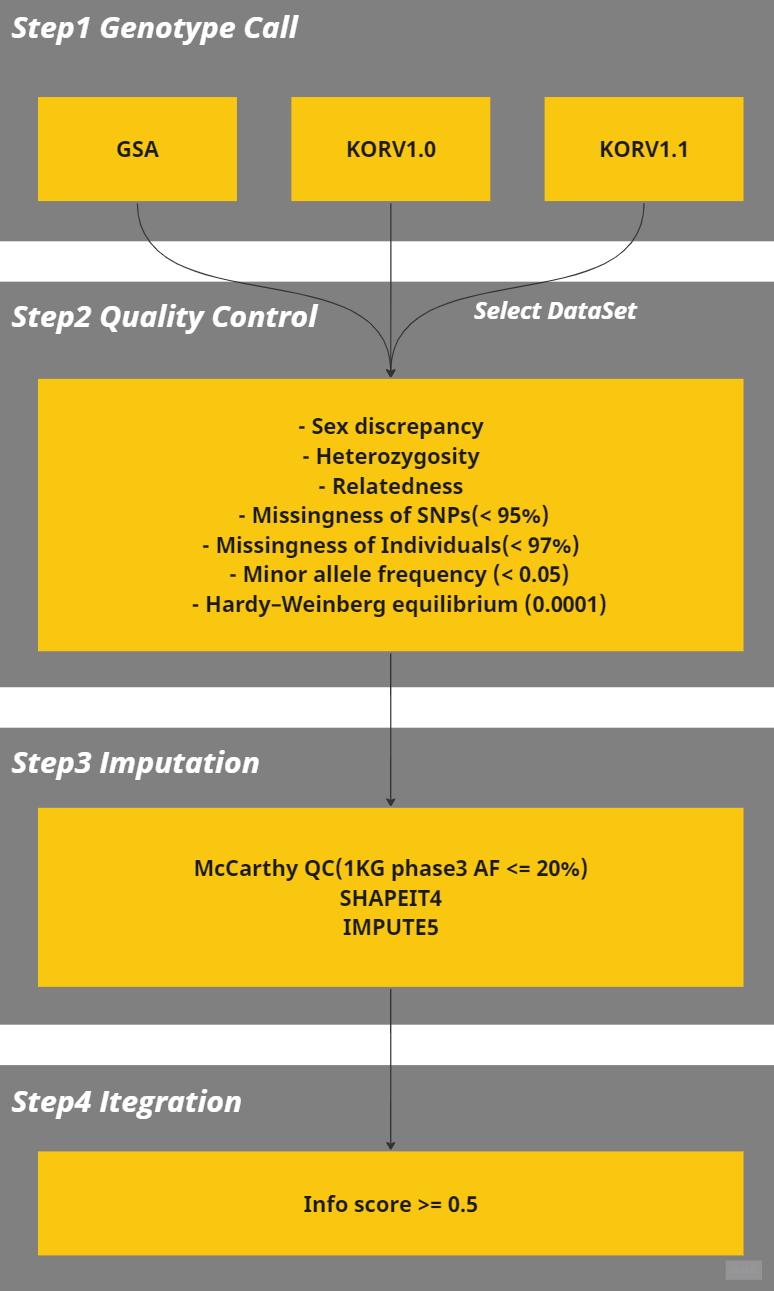
**

Supplement: Supplementary Material 3. — Quality Control process of KCPS-II Biobank [file epih-47-e2025040-Supplementary-3.docx]
