# Supplementary material for: Cohort profile update: the Korean Cancer Prevention Study-II (KCPS-II) biobank
Source: Epidemiol Health. 2025 Jul 29;47:e2025040. doi: 10.4178/epih.e2025040 (PMC12673288; doi:10.4178/epih.e2025040)
Supplement: Supplementary Material 4. — Principal Component Analysis of Non-overlapping Global Screening Array and KoreanChip Subsets [file epih-47-e2025040-Supplementary-4.docx]

**Supplementary Material 4. Principal Component Analysis** **of Non-overlapping Global Screening Array and KoreanChip Subsets**

**
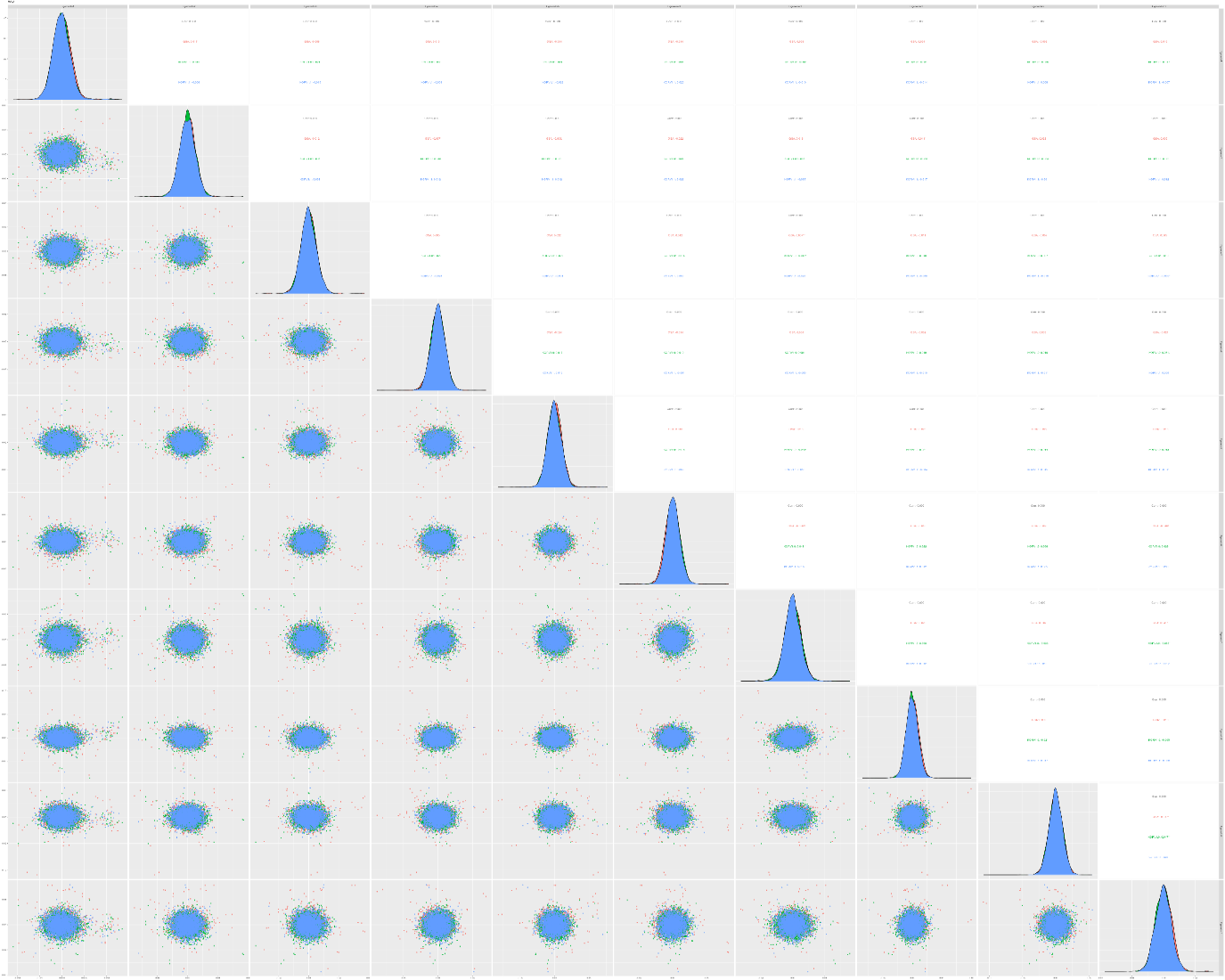
**
