# Supplementary material for: Cohort profile update: the Korean Cancer Prevention Study-II (KCPS-II) biobank
Source: Epidemiol Health. 2025 Jul 29;47:e2025040. doi: 10.4178/epih.e2025040 (PMC12673288; doi:10.4178/epih.e2025040)
Supplement: Supplementary Material 5. — Principal Component Analysis to Assess Batch Effects Between Global Screening Array and KoreanChip Genotyping Arrays [file epih-47-e2025040-Supplementary-5.docx]

**Supplementary Material 5.** **Principal Component Analysis** **to Assess Batch Effects Between Global Screening Array and KoreanChip Genotyping Arrays**

| 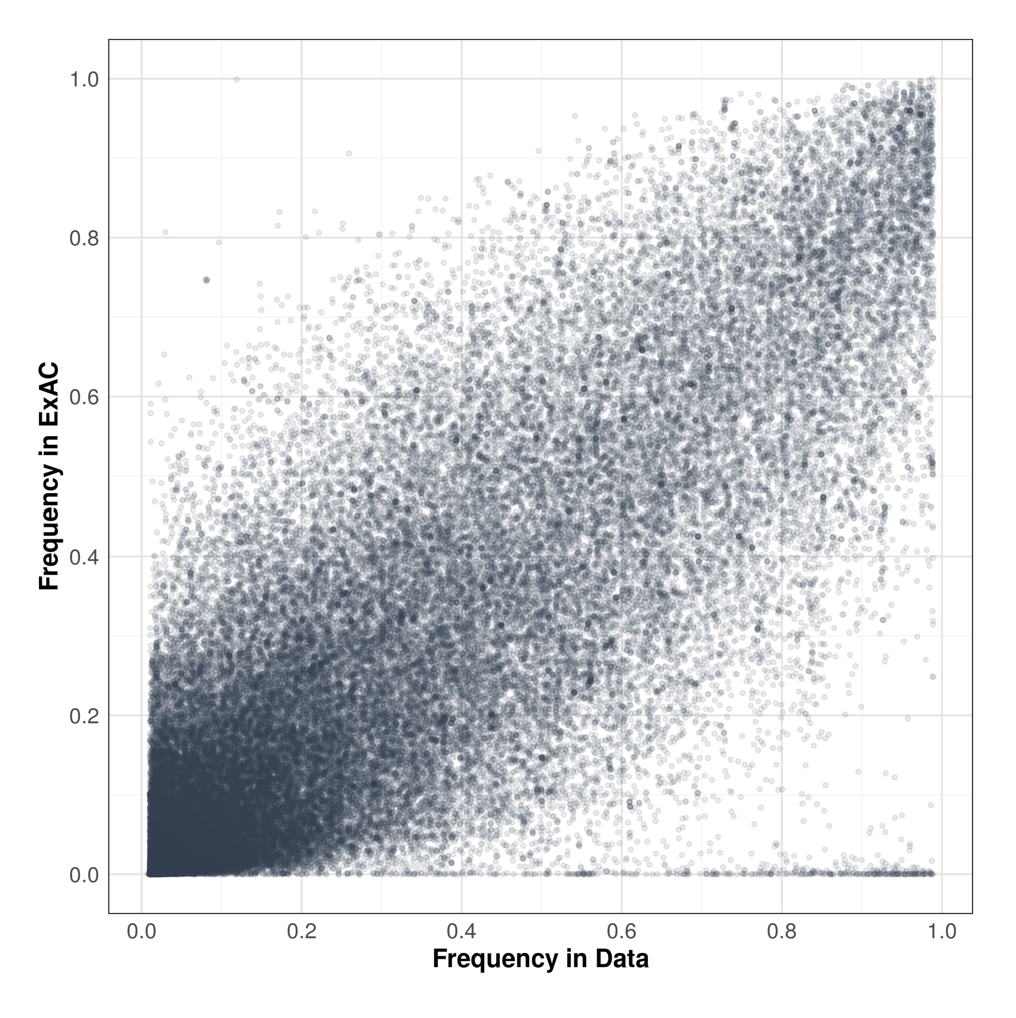 | 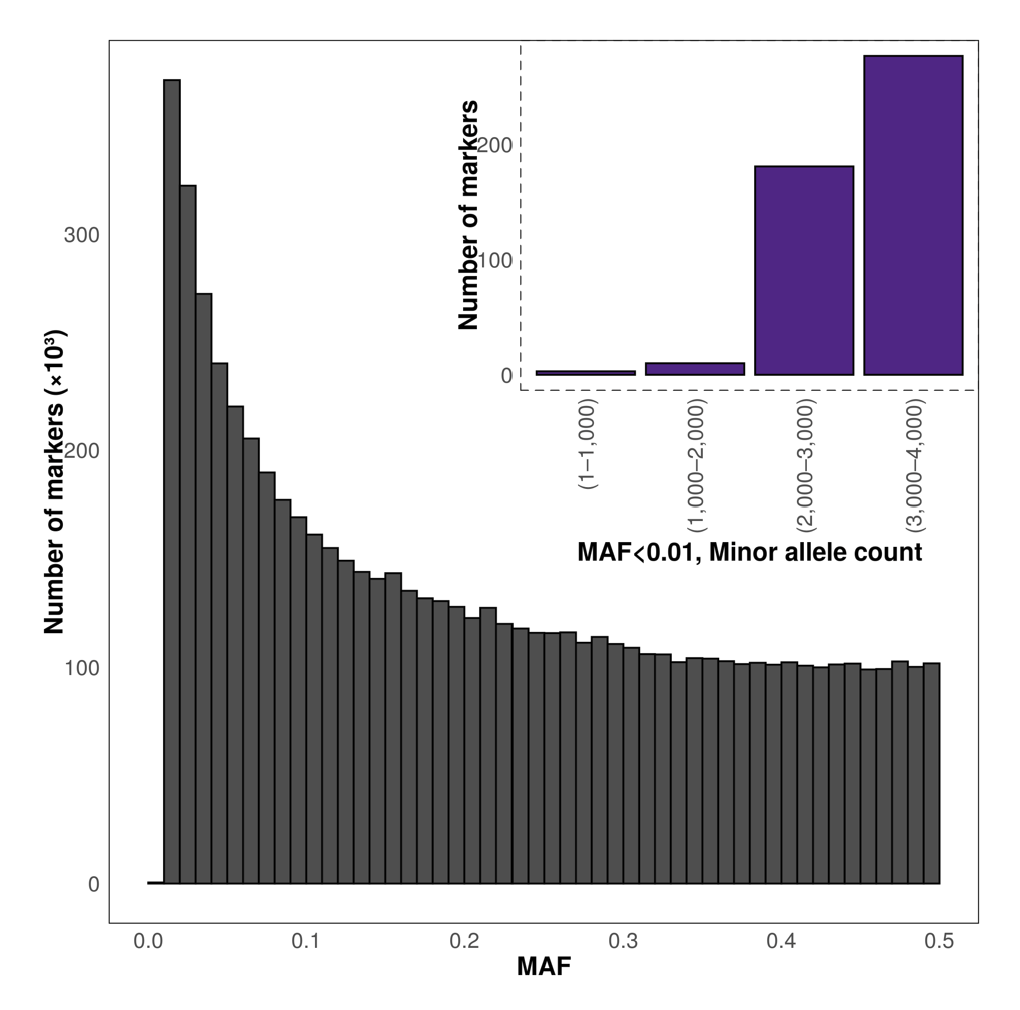 |
| --- | --- |
| The correlation of frequencies between the same markers in KCPS-II and ExAC | The frequency of markers according to MAF. The upper right figure shows the frequency according to the number of MAFs below 1% |

ExAc, Exome Aggregation Consortium; MAF, Minor Allele Frequency
