# Supplementary material for: Cohort profile update: the Korean Cancer Prevention Study-II (KCPS-II) biobank
Source: Epidemiol Health. 2025 Jul 29;47:e2025040. doi: 10.4178/epih.e2025040 (PMC12673288; doi:10.4178/epih.e2025040)
Supplement: Supplementary Material 6. — The list of studies produced from KCPS-II Biobank using genomic or metabolomic data (after 2022) [file epih-47-e2025040-Supplementary-6.docx]

**Supplementary Material 6. The list of studies produced from KCPS-II Biobank using genomic or metabolomic data (after 2022)**

| **No** | **List of studies using genomic and/or metabolite data** | **Topic** | **Classification** |
| --- | --- | --- | --- |
| 1 | Jee et al. 2025  Nature Comm. 2025;16:4935.  Genome-wide association studies in a large Korean cohort identify novel quantitative trait loci for 36 traits and illuminates their genetic architectures | Quantitative trait | Genome-wide association study |
| 2 | Bae et al. 2025  Nutr Diabetes. 2025;15:16.  Association between serum beta-hydroxybutyrate levels and risk of type 2 diabetes mellitus in patients with impaired fasting glucose | Type 2 diabetes mellitus | Metabolomics |
| 3 | Han et al. 2025  Nutr Metab (Lond). 2025;22(1):30.  The Effect of Major Dietary Patterns on Hypertension in Koreans and Dietary Moderation on Risk Groups: A Cross-Sectional Study | Hypertension | Metabolomics |
| 4 | Shin et al. 2024  Epidemiol Health. 2024;46:e2024070.  Causal association between serum bilirubin and ischemic stroke: Multivariable Mendelian Randomization | Ischemic stroke | Mendelian randomization |
| 5 | Chen et al. 2024  Nat Commun. 2024 26;15(1):3557.  Fine-mapping analysis including over 254,000 East Asian and European descendants identifies 136 putative colorectal cancer susceptibility genes. | Colorectal cancer | Genome-wide association study |
| 6 | Chen et al. 2024  Hum Mol Genet. 2024 1;33(4):333-341.  A large-scale microRNA transcriptome-wide association study identifies two susceptibility microRNAs, miR-1307-5p and miR-192-3p, for colorectal cancer risk. | Colorectal cancer | Genome-wide association study |
| 7 | Lee et al. 2024 Epidemiol Health. 2024;46:e2024096. Causal effect of fasting serum glucose on atherosclerotic cardiovascular disease: a multivariable Mendelian randomization | Atherosclerotic cardiovascular disease | Mendelian randomization |
| 8 | Thomas et al. 2023Nat Commun. 2023;14(1):6147.Combining Asian and European genome-wide association studies of colorectal cancer improves risk prediction across racial and ethnic populations. | Colorectal cancer | Genome-wide association study |
| 9 | Fernandez-Rozadilla et al. 2023Nat Genet. 2023;55(1):89-99.Deciphering colorectal cancer genetics through multi-omic analysis of 100,204 cases and 154,587 controls of European and east Asian ancestries | Colorectal cancer | Multi-omic study |
| 10 | Jee et al. 2023Int J Epidemiol. 2023. Jun 6;52(3):796-805.Polygenic Risk Scores for Prediction of breast Cancer in Korean women. | Breast Cancer | Genome-wide association study |
| 11 | Han et al. 2023  Cancer Metab 2023 Dec 5;11(1):23.  Metabolic changes preceding bladder cancer occurrence among Korean men: a nested case control study from the KCPS-II cohort | Bladder cancer | Metabolomics |
| 12 | Han et al. 2023  J Transl Med 2023;21(1):878.  Non-invasive biomarkers for early diagnosis of pancreatic cancer risk: Metabolite genomewide association study based on the KCPS-II cohort | Pancreatic cancer | Metabolite, genome-wide association study |
| 13 | Jung et al. 2023  Epidemiol Health. 2023;45:e2023077. Genetically determined alcohol consumption and cancer risk in Korea | Cancers | Genome-wide association study |
| 14 | Han et al. 2022  Metabolomics 2022;18(8):62.  High serum levels of L-carnitine and citric acid negatively correlated with alkaline phosphatase are detectable in Koreans before gastric cancer onset | Gastric cancer | Metabolomics |
| 15 | Mishra et al. 2022  Nature. 2022;611(7934):115-123.  Stroke genetics informs drug discovery and risk prediction across ancestries. | Stroke | Genome-wide association study |
| 16 | Shu et al. 2022  Cancer Epidemiol Biomarkers Prev. 2022 1;31(6):1216-1226.  Large-scale Integrated Analysis of Genetics and Metabolomic Data Reveals Potential Links Between Lipids and Colorectal Cancer Risk. | Colorectal Cancer | Metabolite, genome-wide association study |
| 17 | Ping et al. 2022  Int J Cancer. 2022 15;151(10):1726-1736.  Developing and validating polygenic risk scores for colorectal cancer risk prediction in East Asians | Colorectal Cancer | Genome-wide association study |
| 18 | Ho et al. 2022  Genet Med. 2022 Mar;24(3):586-600.  Polygenic risk scores for prediction of breast cancer risk in Asian populations. | Breast Cancer | Genome-wide association study |
